# Antibody selection strategies and their impact in predicting clinical malaria based on multi-sera data

**DOI:** 10.1186/s13040-024-00354-4

**Published:** 2024-01-25

**Authors:** André Fonseca, Mikolaj Spytek, Przemysław Biecek, Clara Cordeiro, Nuno Sepúlveda

**Affiliations:** 1 FCT - Faculdade de Ciências e Tecnologia, Universidade do Algarve, Faro, Portugal; 2 CEAUL - Centro de Estatística e Aplicações da Universidade de Lisboa, Faculdade de Ciências, Universidade de Lisboa, Lisbon, Portugal; 3 Faculty of Mathematics & Information Science, Warsaw University of Technology, Warsaw, Poland

**Keywords:** Multivariate Serological Data, Super Learner, Statistical modelling, Malaria outcome prediction, Random forest

## Abstract

**Background:**

Nowadays, the chance of discovering the best antibody candidates for predicting clinical malaria has notably increased due to the availability of multi-sera data. The analysis of these data is typically divided into a feature selection phase followed by a predictive one where several models are constructed for predicting the outcome of interest. A key question in the analysis is to determine which antibodies  should be included in the predictive stage and whether they should be included in the original or a transformed scale (i.e. binary/dichotomized).

**Methods:**

To answer this question, we developed three approaches for antibody selection in the context of predicting clinical malaria: (i) a basic and simple approach based on selecting antibodies via the nonparametric Mann–Whitney-Wilcoxon test; (ii) an optimal dychotomizationdichotomization approach where each antibody was selected according to the optimal cut-off via maximization of the chi-squared (χ^2^) statistic for two-way tables; (iii) a hybrid parametric/non-parametric approach that integrates Box-Cox transformation followed by a t-test, together with the use of finite mixture models and the Mann–Whitney-Wilcoxon test as a last resort. We illustrated the application of these three approaches with published serological data of 36 Plasmodium falciparum antigens for predicting clinical malaria in 121 Kenyan children. The predictive analysis was based on a Super Learner where predictions from multiple classifiers including the Random Forest were pooled together.

**Results:**

Our results led to almost similar areas under the Receiver Operating Characteristic curves of 0.72 (95% CI = [0.62, 0.82]), 0.80 (95% CI = [0.71, 0.89]), 0.79 (95% CI = [0.7, 0.88]) for the simple, dichotomization and hybrid approaches, respectively. These approaches were based on 6, 20, and 16 antibodies, respectively.

**Conclusions:**

The three feature selection strategies provided a better predictive performance of the outcome when compared to the previous results relying on Random Forest including all the 36 antibodies (AUC = 0.68, 95% CI = [0.57;0.79]). Given the similar predictive performance, we recommended that the three strategies should be used in conjunction in the same data set and selected according to their complexity.

## Background

Multi-sera data, where antibodies to multiple antigens are measured in blood samples from the same individual, are becoming widely available in malaria research due to substantial developments at the level of serological assays [1–4]. This public availability has boosted basic research on the discovery of key antibodies associated with protection to malaria [5–10]. It has also motivated the development of serological-based algorithms that could predict not only past exposure to malaria parasites [11, 12], but also time since the last infection [13]. It has been suggested that these algorithms could help design better malaria control strategies, such as the serological testing and treatment (seroTAT) approach based on 8 antibodies for detecting Plasmodium vivax cases that should be targeted to receive an anti-hypnozoite therapy [12].

In these multi-sera studies, the total number of antibody targets varied from dozens [8, 10, 13] to thousands [6, 7, 14]. This number implies a huge computational cost for algorithms that search for the best model for the data. To overcome this problem, a brute-force approach (where every possible antibody combination is tried out) is computationally feasible for no more than 5 antibody targets [8]. However, above that number, implementation of brute-force approaches is not recommended [10, 12]. This computational drawback motivates the use of data analysis strategies that are generically divided into an antibody or feature selection stage, followed by a predictive one, in which several statistical or machine learning models are estimated from the data [7, 9, 10, 13, 15]. In this scenario, the initial antibody selection stage determines the predictive performance of the models to be constructed in the following stage.

Antibody selection can be formulated as the procedure to determine which antibodies are important to predict an outcome of interest [16–18]. However, this selection hides the question whether data transformation, including dichotomization, should be used. Data transformation is particularly relevant in multiplex serological assays, because distinct data distributions can emerge due to differences in the calibration curves across antibodies, as demonstrated in assay-optimization studies [16–18]. Until now, antibody selection has been carried out using only raw or untransformed [5, 6] data or seroprevalence-like data but [10, 12] without any combination of both. Additionally, the transformation of each antibody data is typically not considered. Therefore, current antibody selection procedures for multi-sera data lacks the flexibility to accommodate different data patterns. The current study tackles this issue and shows that it can potentially increase the chance of obtaining improved outcome predictions.

This paper aims at evaluating three feature selection strategies for the identification of antibody responses that could predict clinical malaria with increased accuracy. Initially, we implemented a basic approach where the statistical significance for the nonparametric Mann–Whitney-Wilcoxon test was obtained for each antibody comparing the protected individuals to susceptible ones. A second strategy is also presented in which data of each antibody is initially dichotomized using an optimal cut-off point in the antibody distribution based on the maximization of the χ^2^ test statistic. Finally, we introduced a general parametric strategy for antibody selection in which a combination of transformed and dichotomized antibody data can be selected for the predictive phase. This strategy adds flexibility to feature selection by combining the Box-Cox data transformation with well-known parametric statistical tests.

To illustrate these three strategies, we analyzed a published dataset on Immunoglobulin G (IgG) antibody responses to 36 Plasmodium *falciparum* (*Pf)* antigens in Kenyan children to understand protection to clinical malaria [8] and whose data analysis was previously done with Random Forests [15].

## Methods

### Data under analysis

We re-analyzed published data of 121 Kenyan children (age range: 1–10 years) described in detail elsewhere [8]. All children had a documented parasitaemia (parasite-positive) at the time of sampling and were ﻿monitored for clinical episodes of malaria over a follow-up period of 6 months. As in the original publication, children were considered susceptible (Sus, n_s_ = 40) or protected (Prt, n_p_ = 81) if they had or did not have any clinical episode during follow-up. The serological data referred to individual IgG antibody responses to 36 *Plasmodium falciparum* antigens. These antibody responses were measured by multiple enzyme-linked immunosorbent assays (ELISA). Detailed information about recruitment, study design and experimental protocols, among other aspects of these data, can be found in the original publication [8].

### Preliminary antibody feature selection using random forest

The Random Forest (RF) works by constructing multiple decision trees trained on different parts of the same training set by a resampling process called bootstrap aggregation or bagging [19]. RF were implemented by repeatedly fitting the model to 1000 resampled subsets of the data (100 repeats of tenfold cross-validation). For each repetition, the dataset was divided into 10 folds, of which 9-folds were used to perform an inner tenfold cross-validation [20]. The number of trees to grow and the number of predictors randomly sampled as candidates in each split was set to default [21] (number of trees = 500; number of predictors randomly selected = 2, 19 and 36), and the optimization criterion was the maximization of the area under the Receiver Operating Characteristic (ROC) curve (AUC) [22]. Feature importance was determined by the mean decrease in accuracy [23]. Briefly, for each tree, the prediction accuracy on the out-of-bag portion of the data was recorded. Then, after permuting each predictor variable, the prediction accuracy on the out-of-bag portion of the data was once again recorded. The difference between the two accuracies was then averaged across all the generated trees, and normalized by the standard error [23].

### Antibody selection based on a simple non-parametric approach

The first antibody selection strategy was used to select the antibodies by their statistical significance according to the non-parametric Mann–Whitney-Wilcoxon test comparing the protected and susceptible groups for each antibody [24].

### Antibody selection based on optimal data dichotomization

**Fig. 1:**
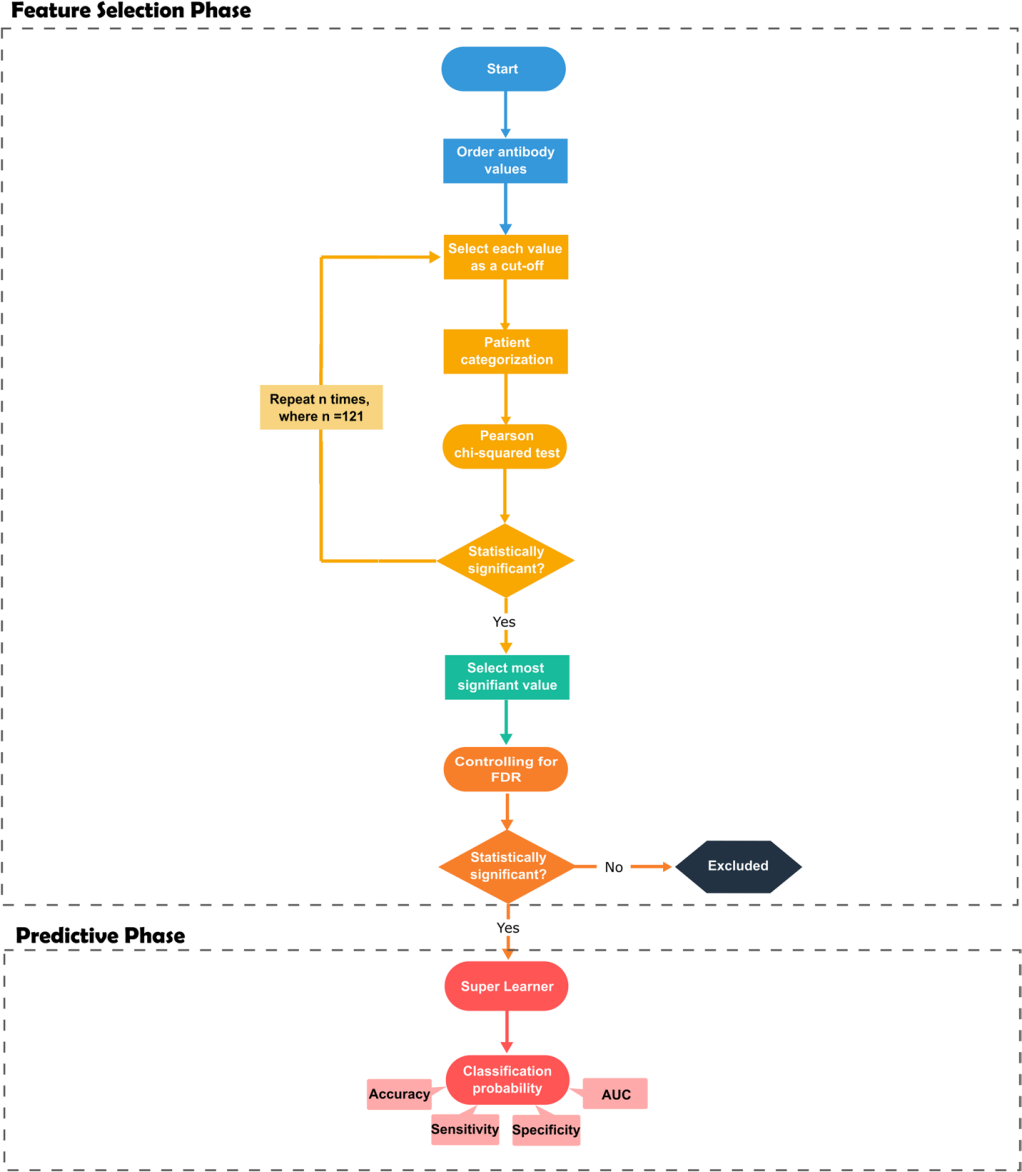
Optimal data dichotomization for antibody selection. The different steps of the analysis are displayed on the workflow using distinct colored shapes. Blue color identifies the beginning of the pipeline where the antibody values are sorted. Light orange identifies the loop for obtaining the χ^2^ test *p*-values for each potential cut−off. Green indicates the selection of the most significant cut-off. Dark orange refers to the assessment of the statistical significance of the most significant cut-off after controlling for the False Discovery Rate (FDR) with the Benjamini−Yekutieli procedure. Red refers to the implementation of the Super Learner and the computation of the classification probability. Additional information is provided by the faded light orange and red colored shapes

The second antibody selection strategy was based on a procedure in which the optimal cut-off to differentiate one study group from another was estimated by maximizing the χ^2^ statistic for testing independence in two-way contingency tables, as done elsewhere [25, 26] (Fig. 1). In more detail, the values of each antibody were sorted by increasing order and then used to divide individuals into two latent serological groups (i.e., seronegative/seropositive individuals or high/low responders). For each value of a given antibody, the resulting data were summarized into a two-way contingency table comprising the qualitative variables serological status (below/above the cut-off) and malaria protection status (protected/non-protected). The χ^2^ test statistic was then calculated for this contingency table. After repeating this procedure for all antibody values, the optimal cut-off was selected as the value that maximized that test statistic, meaning the one that provided the best discriminatory ability between both groups of patients. After selecting the optimal cut-off, we calculated the respective *p-value* associated with the χ^2^ test. The dichotomized data was then used for the predictive phase. This procedure was finally repeated for each of the 36 antibodies included in the dataset. Note that this procedure is conceptually equivalent to predict the outcome with individual decision trees using data of each antibody separately. In this procedure, we also quantified the uncertainty around each optimal cut-off by means of a 95% confidence interval. With this purpose, we used the following Bootstrap algorithm in the respective calculation: (i) generate a new sample (with the same sample size) with replacement from the observed sample of the antibody under analysis; (ii) determine the optimal cut-off value as described above; (iii) repeat points (i) and (ii) 1000 times and saving the respective optimal cut-off values; (iv) determine a 95% confidence interval by calculating the empirical 2.5% and 97.5% quantiles of the Bootstrap samples related to the estimated optimal cutoff values.

### Antibody selection based on a hybrid parametric/non-parametric approach

**Fig. 2:**
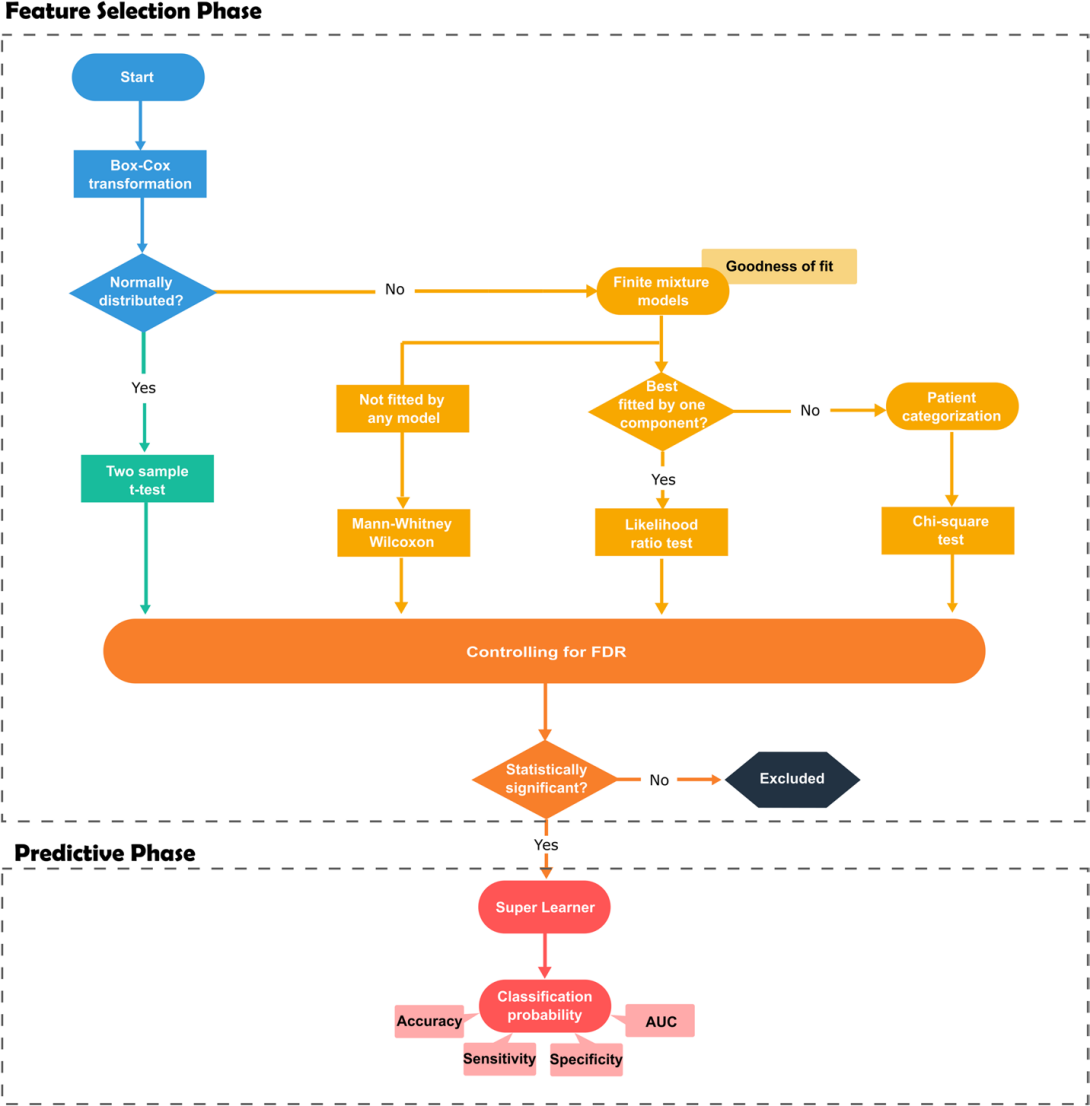
Parametric antibody selection. The different steps of the analysis are displayed on the workflow using distinct colored shapes. Blue color identifies the beginning of the pipeline where the normality assumption is verified after Box−Cox transformation. Green refers to the calculation of the t−test statistic for those antibodies for which the normality assumption was verified. Light orange refers to the implementation of the finite mixture models to those antibodies or which normality assumption failed and implementation of the different tests as according to the best fitted model, or failure to do so. Dark orange refers to the assessment of the statistical significance after controlling for the FDR with the Benjamini−Yekutieli procedure and red to the implementation of the Super Learner and computation of the classification probability. Additional information is provided by the faded light orange and red colored shapes

We adopted an alternative antibody selection approach using different parametric models or statistical tests (Fig. 2). In the first step, we determined the optimal Box-Cox transformation for each antibody. This transformation was sought to obtain normal distributions with homogeneous variances in both groups. We searched the best parameter of this transformation (hereafter denoted as λ) within the interval (-4;4) by maximizing evidence for a Normal distribution using the Shapiro–Wilk (SW) test where the null hypothesis states that the data comes from a normal distribution (with unknown parameters) [27]. A significance level of 5% was specified to assess whether the data of each antibody could follow a normal distribution.

In the antibodies for which there was no evidence against the normal distribution, we calculated the *p-value* for the t-test aiming at comparing the mean values of the susceptible and protected groups. The remaining antibodies, for which there was evidence against the normal distribution, were then evaluated via finite mixture models given that it is recurrent to find latent populations in serological data [28]. Using transformed data, we estimated two-component mixture models based on Normal, Generalized t, Skew-Normal and Skew-t distributions by maximizing the likelihood function via the Expectation–Maximization algorithm [29]. We also estimated the Generalized t, Skew-Normal and Skew-t distributions to assess the evidence that the data could come from a single non-Normal serological population beyond the ones identified by the Box-Cox transformation. We compared all these models using the Akaike’s Information Criterion (AIC) and performed the Pearson’s goodness-of-fit test by dividing the respective data into deciles (i.e., 10%-quantiles). Minimization of the AIC, together with a good fit to the data, at the significance level of 5%, was the criterion for selecting the best model. For antibodies whose data provided evidence of two latent serological populations, we divided the individuals into two latent serological groups using the optimal cut-off by maximization of the χ^2^ statistic (as described in the previous section). In the antibodies for which there was evidence for a single latent serological population antibody, we constructed two linear regression models using the antibody values as the response variable. The first model comprised only the intercept (i.e., not including any covariate), while the second model comprised the malaria protection status as the single covariate. We then computed the *p-value* of the Wilks likelihood ratio test to compare the two models at the significance level of 5%. The rejection of the null hypothesis suggested statistically significant differences between the two models under comparison. Finally, antibodies that could not be fitted by any of the above parametric models were analyzed Mann–Whitney-Wilcoxon test to compare the median values of the protected and susceptible groups.

### Correction for multiple testing

In each antibody selection strategy, all the *p-values* obtained were adjusted to ensure a global false discovery rate (FDR) of 5%. This *p-value* adjustment was made via the Benjamini-Yekutieli procedure under a general dependence assumption between tests [30]. All antibodies with adjusted *p-values* < 0.05 were carried forward to the predictive analysis.

### Predictive stage

When we analyzed data resulting from each antibody selection strategy, we adopted a Super Learner (SL) approach to predict the malaria protection status of each individual [31, 32]. In general, this approach aims to estimate different classifiers whose individual predictions for each study subject are combined into a pooled estimate via a weighted average calculated by cross-validation. To construct this pooled estimator, we used the following 5 classifiers for each set of antibodies selected: logistic regression model (LRM) with main effects only, RF, linear discriminant analysis (LDA), quadratic discriminant analysis (QDA), and extreme gradient boosting (XGB). Note that the inclusion of RF in the SL model assembly algorithm allowed the comparison of the respective results with the previous one based on the same machine learning technique but using all the 36 antibodies as features. For the antibodies selected by optimal dichotomization antibody selection strategy, we did not include LDA and QDA in the SL algorithm because these classifiers are more appropriate for data containing quantitative predictors only.

To assess the quality of the final predictions, we estimated the ROC curve and its area (AUC) [22, 33]. In addition, we calculated the confusion matrices where the rows and columns represented the predicted and the observed status of the individuals, respectively [34]. The predicted values in these confusion matrices were calculated using the point in the ROC curve that minimizes the distance to the point (0,1) related to the perfect classification of the individuals, here called ROC01 criterion [35]. From the standpoint of constructing a fair classifier [36, 37], we also determined the predictive performance by the point in the ROC curve in which sensitivity (protected) and specificity (susceptible) were approximately equal [35]. This criterion is hereafter denoted as SpEqualSe criterion [35].

### Statistical software

All statistical analyses were implemented in the R software [38] version 4.3.0 using the following packages: “AID” to perform Box-Cox transformation and to perform the Normality tests [39]; “caret” to construct the confusion matrices[23]; “doParallel” for parallel processing and faster run times [40]; “dplyr” to better manipulate the data [41]; “ggplot2” to plot the data [42]; “ggrepel” to avoid overlaid text on plots [43]; “lmtest” to perform the likelihood ratio test [44]; “MASS” for general analysis [45]; “mixsnsm” to estimate mixture models based on Skew-Normal and Skew-t distributions [46]; “OptimalCutpoints” to obtain the point in the ROC curve that minimizes of the distance to the point (0,1) [35]; “pROC” to estimate ROC curves [47]; “sn” to perform linear regression models based on Skew-Normal or Skew-t distributions for the residuals [48]; “SuperLeaner” to perform all the predictive analysis [31]; “tydir” to facilitate data manipulation [49].

## Results

### Preliminary analysis based on the random forest approach

**Fig. 3:**
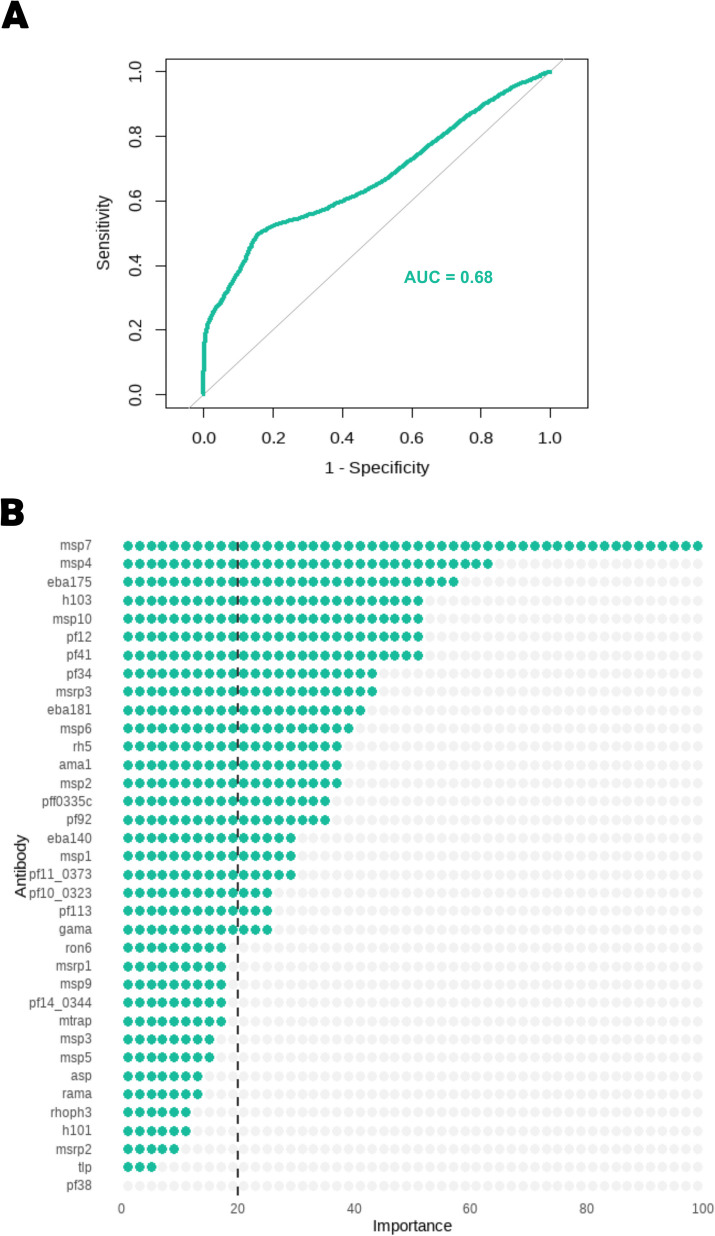
Analysis of an RF using all the 36 antibodies as features. **A **ROC curve and its AUC; **B**) Estimated importance of each antibody in the RF

Initially, an RF model was implemented using all the 36 antibodies as features in order to replicate the results previously reported by Valleta and Recker [15]. We were able to reproduce the previously reported AUC of 0.68 (95% CI = (0.57;0.79)) (Fig. 3A). Looking at the feature importance values, we concluded that all except one of the 36 antibodies were required to achieve this predictive performance (Fig. 3B). Nevertheless, a more thorough analysis of the feature’s importance values reveals that several features had very low importance values (below 20% importance) (Fig. 3B). This led us to hypothesize that removing these features could improve the model’s performance. Therefore, three distinct *filter* strategies for feature selection were used.

### Analysis based on the simple antibody selection approach

**Fig. 4:**
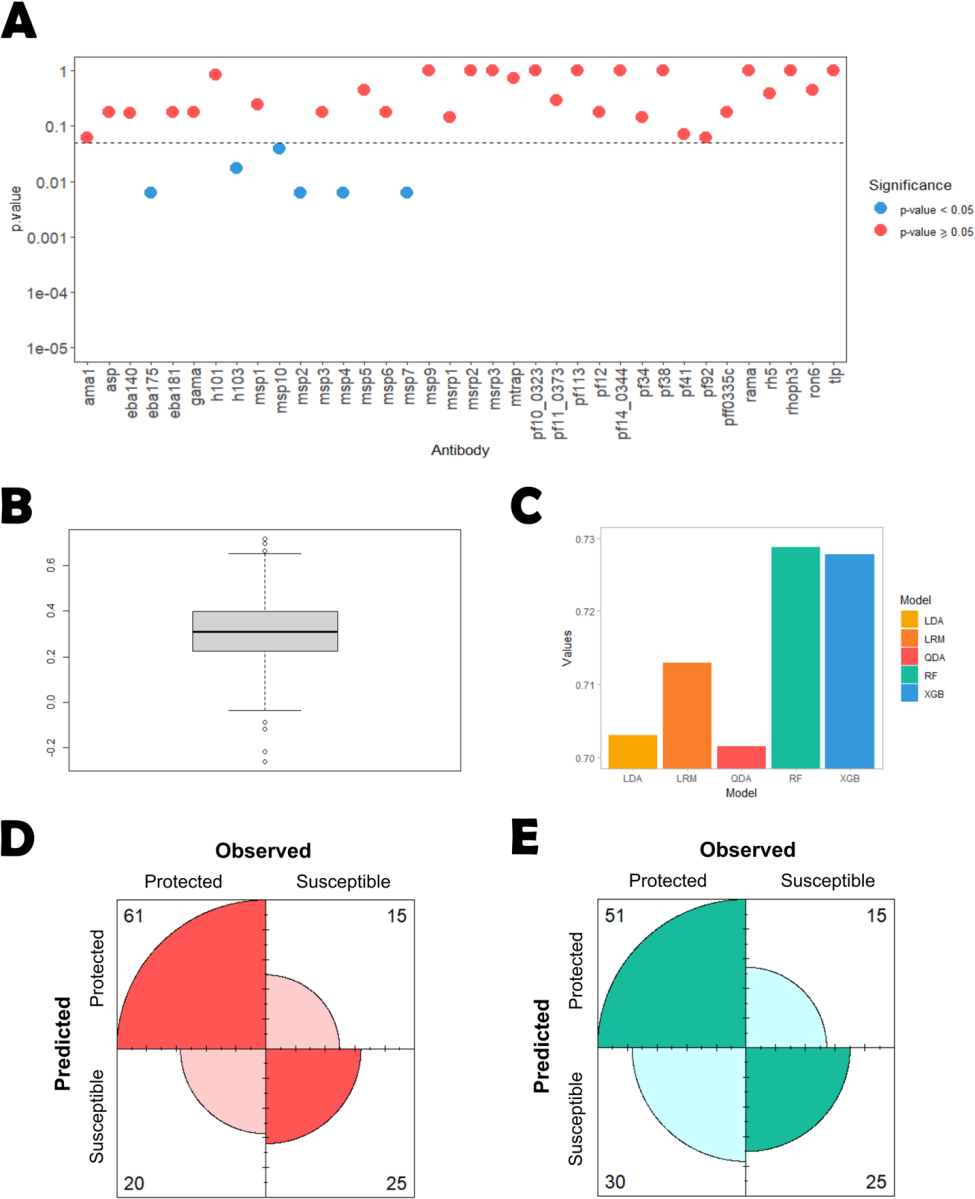
Simple antibody selection results. **A **Statistical significance of each antibody according to Mann–Whitney−Wilcoxon where the p−values were adjusted for an FDR of 5%. **B **Average Spearman’s correlation concerning all the 36 antibodies. **C **Average AUC estimated for each individual model embedded in the Super Learner. **D **Confusion matrix of the predicted versus observed individual’s classification derived from the Super Learner model using the ROC01 and **E**) SpEqualSe criterion

We first tested whether levels of each antibody were significantly different between susceptible and protected individuals using the Mann–Whitney-Wilcoxon test. According to this nonparametric test, 21 out of the 36 antibodies were found statistically significant before adjusting for multiple testing. This number dropped to 6 after controlling for an FDR of 5%: *msp2*, *msp4*, *msp10*, *eba175*, *msp7*, and *h103* (Fig. 4A). This substantial reduction in the number of significant antibodies is likely to be explained by the positive correlation among different antibodies (average Spearman’s correlation coefficient = 0.312; Fig. 4B).

We then constructed a Super Learner classifier based on the data of these 6 antibodies. The average estimates for the AUC were 0.713, 0.703, 0.702, 0.729 and 0.728 using LRM, LDA, QDA, RF and XGB, respectively (Fig. 4C). A closer examination of the RF’s performance (AUC = 0.729) reveals an AUC increment over its performance prior to feature selection (Fig. 3A).

The average weights of these classifiers were 0.089, 0.506, 0.035, < 0.001, and 0.370 in the final predictions, respectively. These weights implied an AUC of 0.719 (95% CI = [0.615, 0.824]) for the SL predictions. Moreover, the SL predictions had a sensitivity of 0.753 and a specificity of 0.625 according to the ROC01 criterion (Fig. 4D). A higher number of protected individuals in the dataset could explain the fact that sensitivity was estimated at a higher value than specificity. To assess the final classifier without this potential selection bias, we determined the point at which the ROC sensitivity and specificity were similar and used it to obtain a fair classification (SpEqualSe criterion). The balanced sensitivity and specificity estimates were 0.630 and 0.625, respectively (Fig. 4E).

### Analysis based on the data dichotomization approach

**Fig. 5:**
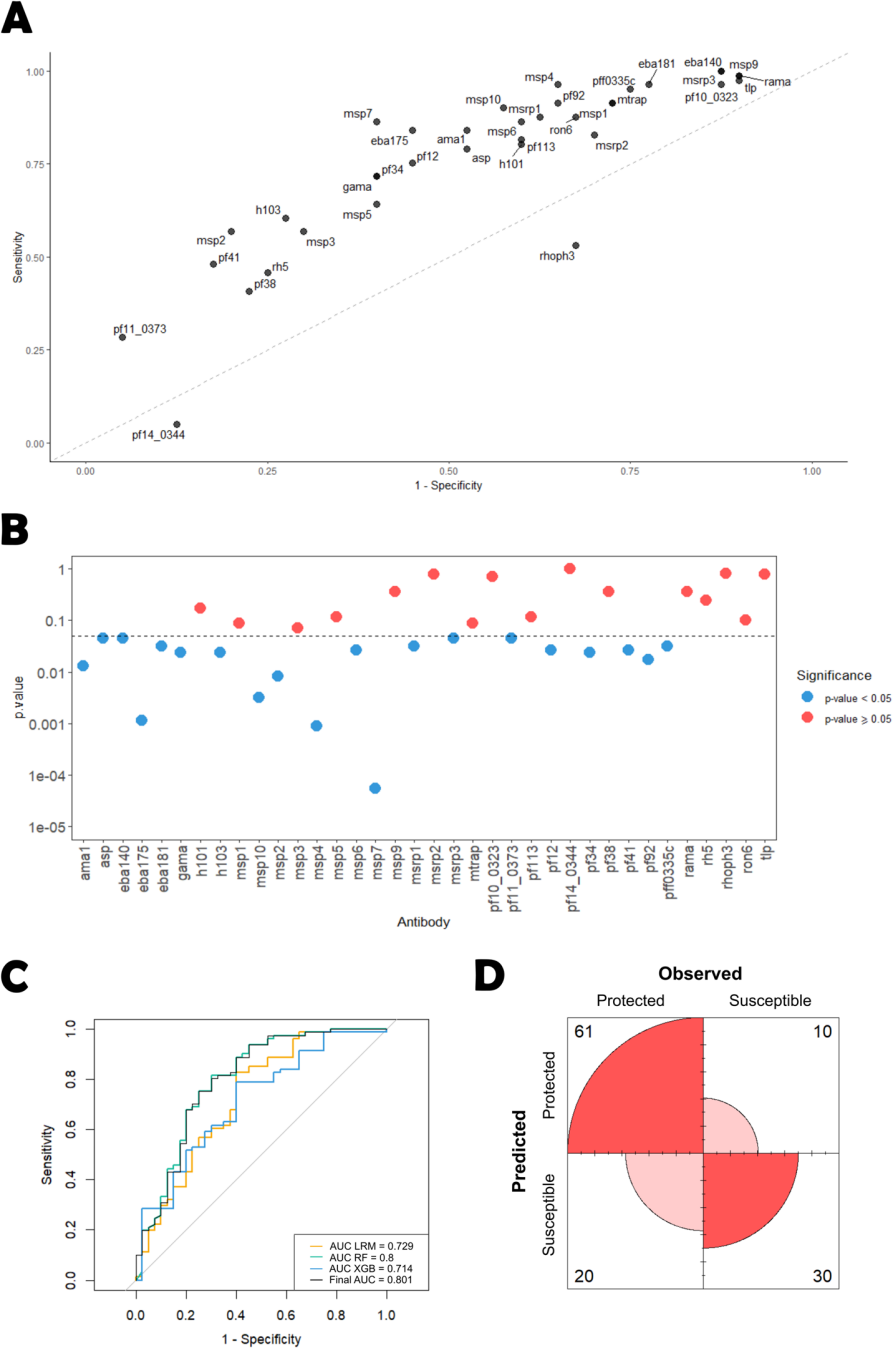
Optimal data dichotomization antibody selection results. **A **Sensitivity versus specificity plot for each antibody according to the cut−off that maximized the Pearson’s χ2 statistic. **B **Statistical significance of each antibody following p−value correction using the Benjamini−Yekutieli procedure. **C **AUCs for the individual models: Logistic regression (LRM), Random Forest (RF) and XGBoost (XGB) embedded in the Super Learner; and the overall AUC provided by the Super Learner. **D **Confusion matrix of the predicted versus observed individual’s classification derived from the Super Learner model using the ROC01 and **E**) SpEqualSe criterion

In this analysis, we determined the optimal classification cut-off for each antibody according to the χ^2^ statistic. The sensitivity estimates using these optimal cut-offs varied from 0.049 (*pf14_0344*) to 1 (*eba140*, *msrp3*), while the specificity varied from 0.100 (*msp9*) to 0.95 (*pf11_0373*). The top 3 antibodies whose optimal cut-offs provided the sensitivity and specificity estimates closest to perfect classification (i.e., specificity = sensitivity = 1) were *msp7* (Se = 0.852, Sp = 0.600), *eba175* (Se = 0.827, Sp = 0.550), and *msp2* (Se = 0.556, Sp = 0.800; Fig. 5A).

There were 28 out of 36 antibodies whose proportions above the respective optimal cut-off were significantly different between protected and susceptible individuals at the 5% significance level (Table 1). The uncertainty around each optimal cut-off was highly heterogenous across these 28 antibodies. On the one extreme, the shortest 95% confidence for the optimal cut-off was obtained for the antibodies against *ron6* (95% CI = [0.04;0.11]). On the other extreme, the widest 95% confidence for the optimal cut-off was obtained for the antibodies against *eba175* (95% CI = [0.10;1.81]). After controlling for an FDR of 5%, the number of statistically significant antibodies dropped to 20 (Fig. 5B). The optimal dichotomization of these antibodies was used in the predictive analysis.

**Table 1:** Results from the 28 antibodies deemed significant by the data dichotomization approach. The antibody levels that maximized the separation between the susceptible and protected group of individuals (Cut-off) and the proportion of seropositive individuals for all (Total), Protected (Prt) and susceptible (Sus) children, respectively

Antibody	P-value	Cutoff (95% CI)	Total	Prt	Sus
*msp1*	0.01	0.14 (0.04;0.99)	0.85	0.91	0.73
*msp2*	< 0.01	0.07 (0.04;0.34)	0.45	0.57	0.20
*msp4*	< 0.01	0.13 (0.10;1.36)	0.86	0.96	0.65
*msp5*	0.02	0.09 (0.06;0.23)	0.56	0.64	0.40
*msp10*	< 0.01	0.25 (0.11;1.57)	0.79	0.90	0.58
*pf12*	< 0.01	0.10 (0.07;0.45)	0.65	0.75	0.45
*pf92*	< 0.01	0.11 (0.05;1.32)	0.83	0.91	0.65
*pf34*	< 0.01	0.07 (0.05;0.15)	0.61	0.72	0.40
*pf113*	0.02	0.05 (0.04;0.13)	0.74	0.81	0.60
*gama*	< 0.01	0.05 (0.04;0.11)	0.61	0.72	0.40
*ama1*	< 0.01	0.16 (0.04;1.09)	0.74	0.84	0.53
*eba175*	< 0.01	0.14 (0.10;1.81)	0.71	0.84	0.45
*eba140*	< 0.01	0.11 (0.11;1.55)	0.96	1.00	0.88
*eba181*	< 0.01	0.11 (0.09;1.46)	0.90	0.96	0.78
*mtrap*	0.01	0.05 (0.04;0.12)	0.85	0.91	0.73
*asp*	< 0.01	0.08 (0.07;0.15)	0.70	0.79	0.53
*msp3*	0.01	0.08 (0.04;0.30)	0.48	0.57	0.30
*msp6*	< 0.01	0.12 (0.10;0.32)	0.78	0.86	0.60
*msp7*	< 0.01	0.24 (0.10;1.27)	0.71	0.86	0.40
*msrp1*	< 0.01	0.05 (0.05;0.22)	0.79	0.88	0.63
*msrp3*	< 0.01	0.04 (0.04;0.10)	0.96	1.00	0.88
*h101*	0.03	0.05 (0.04;0.11)	0.74	0.80	0.60
*h103*	< 0.01	0.07 (0.04;0.24)	0.50	0.60	0.28
*pf41*	< 0.01	0.12 (0.04;0.53)	0.38	0.48	0.18
*pff0335c*	< 0.01	0.05 (0.04;0.35)	0.88	0.95	0.75
*rh5*	0.04	0.16 (0.09;0.25)	0.39	0.46	0.25
*ron6*	0.02	0.04 (0.04;0.11)	0.81	0.88	0.68
pf11_*0373*	< 0.01	0.08 (0.05;0.14)	0.21	0.28	0.05

The AUC of the SL-based predictions was estimated at 0.801 (95% CI = [0.709, 0.892]) (Fig. 5C), which showed an improvement from the previous analysis using a non-parametric antibody selection. The average AUC (and weights) estimates for each classifier were: LRM -0.729 (< 0.001), RF -0.800 (0.973), and XGB -0.714 (0.026). This result showed that, notwithstanding the reasonable AUC estimates for LRM and XGB, the final predictions were basically derived from the RF classifier. Not only that, but the RF’s AUC also increased significantly when compared to implementation using all the variables, highlighting the value of feature selection. Moreover, note that LDA and QDA were not included in the SL algorithm, as they are more suitable for analyzing quantitative multivariate data.

According to the ROC01, the final sensitivity and specificity were estimated at 0.753 and 0.750, respectively. These estimates were identical for the SpEqualSe criterion. In conclusion, this analysis produced a combined classifier that exhibited an improved and better-balanced predictive performance than the previous one. However, this classifier had the disadvantage of including a higher number of antibodies compared to the previous one (20 antibodies versus 6 antibodies).

### Analysis based on the hybrid parametric/non-parametric approach

**Fig. 6:**
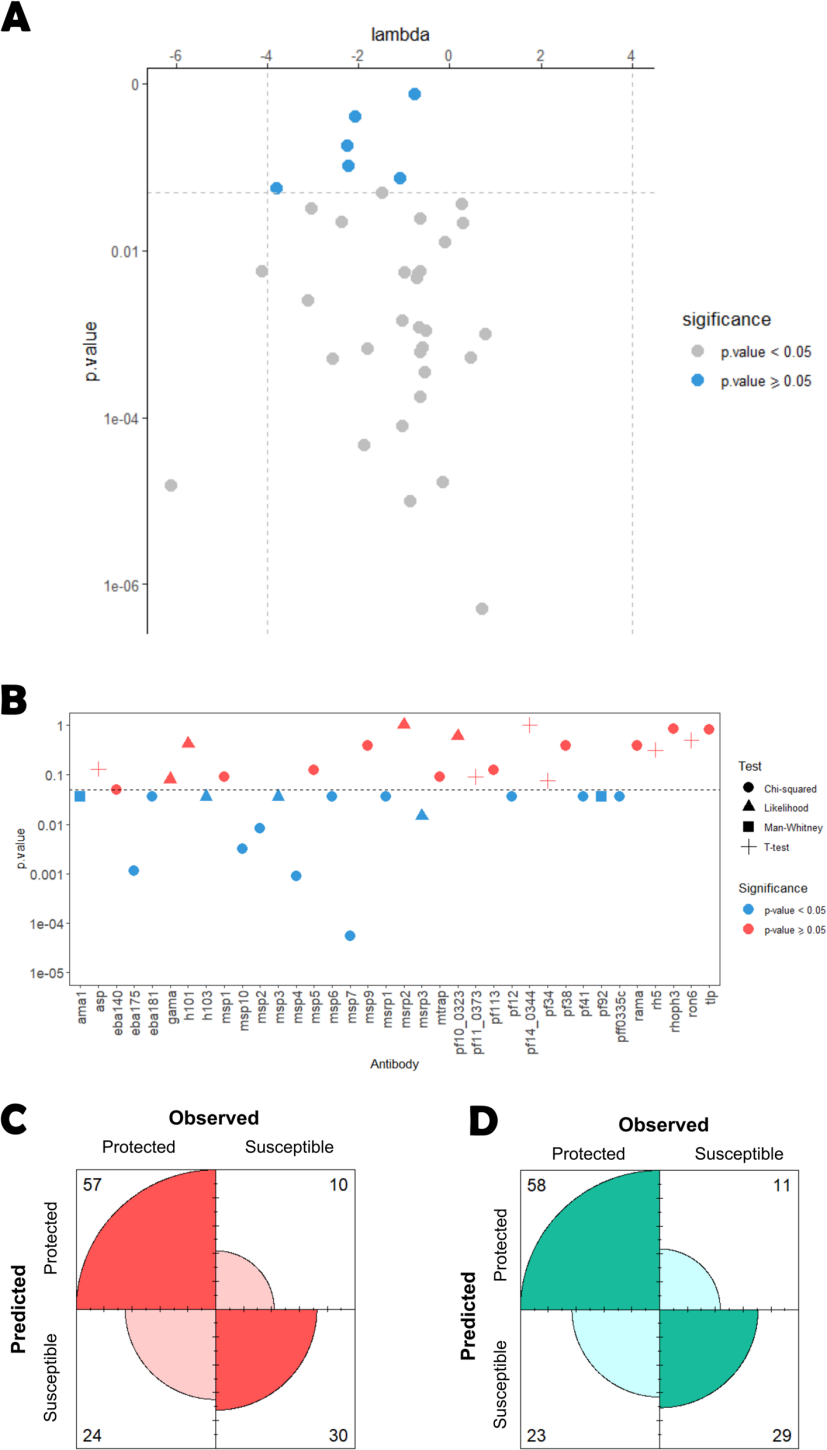
Hybrid antibody selection results. **A** *P*-values for the SW normality test (y-axis) after Box-Cox transformation with the respective lambda (x-axis). **B **Statistical significance of each antibody following *p*-value correction using the Benjamini-Yekutieli procedure. **D **Confusion matrix of the predicted versus observed individual’s classification derived from the Super Learner model using the ROC01 and **D**) SpEqualSe criterion

We first estimated the Box-Cox optimal data transformation and applied it to the antibody data. Then, we compared the protected and susceptible groups using the parametric t-tests for two independent samples. Our findings suggested that there were 6 antibodies whose data in each study group could be analyzed by these tests after the Box-Cox transformation: *asp*, *pf11_0373*, *pf14_0344*, *pf34*, *rh5,* and *ron6* (Fig. 6A)*;* note that, at this stage, we did not adjust the *p-values* of the respective goodness-of-fit tests due to multiple testing, because such adjustment would increase the evidence for the null hypothesis of these tests. In these antibodies, the estimates for the parameter λ of the Box-Cox transformation varied from -3.80 (*ron6*) to -0.78 *(pf34)*.

The estimates suggest that the logarithmic transformation would not be the best to generate a normal distribution. The strongest evidence for a Normal distribution was found for *pf34* with a *p-value* of 0.75 using the SW test (Fig. 6A). The remaining 30 antibody data were then analyzed by fitting finite mixture models based on Normal, Generalized T, Skew-normal, and Skew-T distributions; note that Normal and t distributions come as special cases of the latter probability distributions. For the statistical convenience of having these antibodies defined in terms of positive and negative values, we log-transformed the respective antibody data.

We found evidence that data from 7 antibodies could be described well by either Skew-Normal (*msp3* and *h103*) or Skew-t (*gama, h101*, *msrp2*, *msrp3*, and *pf10_0323*) distributions (Table 2). In this case, the comparison between study groups was made via regression models using these distributions for the errors. Except for the antibodies against *pf92* and *ama1,* data of the remaining antibodies were best described by a mixture of two Normal distributions (4 antibodies), two Skew-Normal distributions (16 antibodies) or two Skew-t distributions (1 antibody; see Table 2). The best fit of these mixture models was obtained for the antibody against *pf113* using a two-component Normal mixture model (*p* = 0.73, Pearson’s goodness-of-fit test; Table 2). For these antibodies, we assumed the existence of a seronegative and a seropositive population. We dichotomized the respective data using the optimal cut-off by maximization of the χ^2^ test statistic. Data of the antibodies against *pf92* and *ama1* could not be fitted by either the Normal distribution after Box-Cox transformation or using the above mixture models. Therefore, we used the Mann-Whitney-Wilcoxon test as the last resort statistical test to compare the protected and susceptible groups. Thus, comparing the protected and susceptible groups using the different tests led to 25 significant antibodies before applying a multiple testing correction. This number decreased to 16 after ensuring an FDR of 5%. These antibodies were found to be significant by the Wilks likelihood ratio test (*msp3*, *msrp3* and *h103*), the χ^2^ test (*eba175*, *eba181*, *msp2*, *msp4*, *msp6*, *msp7*, *msp10*, *msrp1*, pf12, *pf41*, *pff0335c*) and the Mann–Whitney-Wilcoxon test (*pf92, ama1*) (Fig. 6B). In the predictive analysis, data of each antibody were included in the SL approach according to the suggested scale by the antibody selection procedure: log-transformed data for antibodies coming from the Wilks likelihood ratio test, dichotomized seropositive/seronegative data for antibodies coming from the χ^2^ test, and the original scale for the *pf92* and *ama1*-related antibodies coming from the Mann–Whitney-Wilcoxon test. Before obtaining the combined predictions, we checked each individual classifier’s performance. The average AUC were 0.756, 0.807, 0.768, 0.656 and 0.643 using LRM, RF, LDA, QDA, and XGB, respectively. Therefore, the best individual classifier was the RF, which once more performed better than the one implemented prior to feature selection. The average weights of these classifiers were 0.021, 0.912, 0.0132, 0.053, and 0 in the final predictions, respectively, resulting in an AUC of 0.79 CI = [0.7, 0.879]) According to the ROC01 criterion, the sensitivity and specificity were estimated at 0.703 and 0.750, respectively (Fig. 6C). Moreover, based on the ROC curve, the best balance between these quantities was obtained for a sensitivity and a specificity of 0.716 and 0.725, respectively (Fig. 6D).

**Table 2:** Analysis based on finite mixture model. Results from the analysis of 28 antibodies based on finite mixture models, where AIC and GOF denote the Akaike’s information criterion and the Pearson’s goodness-of-fit test, respectively

Antibody	Best Mixture Model	# Components	AIC	P-value (GOF)
*eba140*	Skew Normal	2	23,92	0,32
*eba175*	Skew Normal	2	33,29	0,03
*eba181*	Skew Normal	2	42,9	0,03
*gama*	Skew-t	1	-272,19	0,24
*h101*	Skew-t	1	-230,91	0,33
*h103*	Skew Normal	1	-41,91	0,72
*msp1*	Skew Normal	2	25,35	0,26
*msp10*	Normal	2	71,52	0,07
*msp2*	Skew Normal	2	-24,09	0,43
*msp3*	Skew Normal	1	1,46	0,32
*msp4*	Skew Normal	2	76,23	0,04
*msp5*	Normal	2	-71,25	0,33
*msp6*	Normal	2	-168,02	0,35
*msp7*	Skew Normal	2	46,11	0,16
*msp9*	Skew Normal	2	-10,75	0,53
*msrp1*	Skew-t	2	-89,1	0,06
*msrp2*	Skew-t	1	-122,32	0,12
*msrp3*	Skew-t	1	-283,83	0,02
*mtrap*	Skew Normal	2	-213,58	0,13
*pf10_0323*	Skew-t	1	-344,51	0,62
*pf113*	Normal	2	-139,5	0,73
*pf12*	Skew Normal	2	-33,29	0,24
*pf38*	Skew Normal	2	99,41	0,05
*pf41*	Skew Normal	2	35,96	0,10
*pff0335c*	Skew Normal	2	4,83	0,04
*rama*	Skew Normal	2	-153,54	0,32
*rhoph3*	Skew Normal	2	-152,73	0,02
*tlp*	Skew Normal	2	-426,93	0,02

## Discussion

Multi-sera data, where thousands of antibody targets are simultaneously measured, can increase the chance of discovering the antibodies responsible for natural protection against malaria or the antibodies that can be used to detect previously exposed individuals to malaria parasites [50–52]. Nonetheless, this type of data brings novel challenges [53, 54]. One of the main drawbacks when dealing with this type of data is the difficulty of identifying the relevant features for the task at hand. Among the thousands of features screened, most will be irrelevant or redundant and will negatively impact the predictive ability of a predictive model [55]. Not only that, trying to fit a predictive model with many features increases the computational complexity and cost, reduces the model generalization ability, and affects the interpretability of the model [54]. To overcome these limitations, feature selection strategies have been proposed, where the aim is to identify and remove all the irrelevant features so that the learning algorithm focuses only on those features of the training data useful for prediction [53]. This leads to not only a simpler interpretability, as when a small number of features is selected, their biological relationship with the target disease is more easily identified, but also a lower computational cost and increased accuracy stemmed from reducing the chance of overfitting [54, 56]. Therefore, feature selection before the implementation of a predictive model is strongly advocated [57]. Amongst the different feature selection approaches, we opted for the use of *filter* methods in this study [53, 56, 57]. These rely on statistical measures (i.e., *p-value,* correlation coefficient), and their application precedes the predictive phase, thus being independent of any predictive model [56, 57]. For this reason, they are usually very fast to implement. Here we will discuss the advantages and drawbacks of the distinct filter methods employed in each proposed methodology. The simple approach relying on the Mann–Whitney-Wilcoxon test for feature selection is the most scalable approach for larger datasets among the ones here proposed. It is the most straightforward and fastest approach to implement, making it an appealing tool for those looking for a low complexity model when conducting a classification task. Moreover, given its ranking intrinsic nature, this strategy represents the best option to achieve reproducible results [24]. Nevertheless, its low statistical and computational complexity comes at a cost since this feature selection approach might lead to a lower predictive performance when compared to the other strategies, as demonstrated in this study.

The best predictive performance was obtained from the feature selection strategy based on data dichotomization. This performance contradicts the general expectation of losing statistical information every time one analyses dichotomized data [58–60]. However, in serological data analysis, one typically expects the existence of a single latent seronegative population and a single latent seropositive population in a given antibody distribution [28, 61, 62]. These populations can be conceptually interpreted as noise and signal of genuine antibody responses to a given antigen, respectively. In this scenario, data dichotomization is a natural way to separate noise from a true biological signal. In other words, data dichotomization comes naturally if one intends to eliminate the effect of noise in the respective data analysis. In fact, the original study reported that the seroprevalence varied from 5 to 96% in the dataset analyzed [9]. Hence, all the antibodies contained some degree of noise in the respective data and the presence of such a noise across multiple antibodies is a likely explanation for the best performance of this feature selection method in the dataset analyzed. In the same line of thought, we speculate that a better predictive performance using this feature selection strategy could not be achieved due to a possible overlap between seronegative and seropositive populations. The detailed exploration of this point, although interesting, was beyond the scope of the present study.

The data dichotomization approach also showed a great practical advantage due to its simple computational implementation. However, the performance of this approach might be dependent on the uncertainty around the optimal cut-off for each antibody. As demonstrated by our analysis, this uncertainty varied substantially from one antibody to another. Such a variation is likely to be explained by not only a relatively small sample size of the original study, but also the ratio between the proportions of susceptible and resistant individuals. Thus, the cut-offs here reported should be used with caution. Ideally, they should be confirmed with a larger data set where there is a good balance between susceptible and resistant individuals.

Notwithstanding being more complex from a statistical standpoint, our hybrid approach provides a more comprehensive analysis of the data. In this approach, feature selection is made on the basis of data transformation and dichotomization via mixture modelling, thus accommodating different data patterns. However, this feature selection strategy is expected to increase the computational time dramatically as the number of antibodies under analysis increases. The computational implementation in user-friendly packages is also not trivial in relation to the other feature strategies applied in this study. Finally, this feature selection strategy is based on complex statistical models such as finite mixture models related to Skew-Normal distributions. In this scenario, this strategy seems less appealing to the malaria research community where, despite the efforts to improve mathematical modelling capacity, the availability of qualified staff with statistical and machine learning skills remains scarce. Therefore, the use of simple filter methods seems a more viable solution at the moment, especially, when it comes to analyzing data featuring thousands of antibodies. Such a case is seen in Proietti et al. [7] where antibodies with a *p*-value < 0.01 for the univariate logistic regression were selected after Bonferroni correction followed by sparse partial least squares discriminant analysis (sPLS-DA) and Support Vector Machine (SVM). Another example is the use of the Spearman’s correlation coefficient to remove highly correlated antibodies prior to the implementation of the RF presented by Valletta and Recker [15].

A significant disadvantage of *filter* methods is the inability to detect complex relations between multiple features and the outcome of interest, which generally translates into poorer results in the predictive phase [56, 57]. Thus *wrappers* or *embedded* methods are more appealing. *Wrappers* are created around a particular classifier and rely on the classifier’s information concerning feature relevance [56, 57]. For this reason, the computational effort they require is usually significant, becoming unfeasible in real time when thousands of features are considered. Therefore, *wrappers* are often avoided, and their implementation for feature selection in malaria is scarce [8]. A more attractive approach are *embedded* methods that use the core of a classifier to establish a criterion to rank features [53, 56]. *Embedded* algorithms perform feature selection during the classifier training procedure while optimizing the feature set used to achieve the best accuracy. Therefore, they are less computationally costly than wrappers while still dealing with the complex interactions between multiple features and the outcome [53, 56]. Examples of *embedded* feature selection methods intending to unveil antibody immune signatures in malaria are described in the literature. Aitken et al. [63] used an elastic net-regularized logistic regression for antibody selection followed by a partial least squares discriminant analysis to find a minimal set of antibodies that accurately classified the individuals under analysis. Helb et al. [13] used a hierarchical criterion for feature selection, where a combination of *embedded* and *filter* methods was performed before the implementation of a Super Learner for predicting past exposure to malaria. Here, the Least Absolute Shrinkage and Selection Operator (LASSO) regression was initially used to select one third of the responses. Then, using variable importance measures from RF, they iteratively selected the best responses which were then ranked by the *p-values* for the underlying Spearman’s correlation coefficient [13]*.* Although not implemented here due to the relatively small number of features, we envision that *embedded* feature selection approaches will be more useful in datasets in where the number of antibody responses exceeds the number of observations, as already seen in a study from Mali [14]. A forthcoming research study will investigate this solution and its impact on variable selection.

Alternative approaches to feature selection techniques for identifying the optimal antibody combinations for the task at hand have also been proposed [10, 12]*.* These rely on simulated annealing algorithms that efficiently explore the vast space of feature combinations and thus identify the optimal feature combination solution given a fixed number of features defined by the user [10]*.* Whether this approach is preferable over feature selection techniques is an interesting research question for future work.

Concerning our predictive analysis, we adopted a SL approach. The reasoning for this option relied on the fact that by combining the individual predictions of each classifier, the SL avoids the bias created by manually choosing the best-fitting model procedure and often provides better results than each individual classifier [31, 32]. However, this was not always the case, as the RF alone tended to provide better predictions than the SL. Given that RF is an *embedded* method, it performs feature selection during the classifier training procedure and thus we speculated that the removal of further features could be behind this increased performance [20, 64]. Nevertheless, our validation analysis revealed that regardless of the strategy chosen for feature selection, nearly all features were important for classification purposes. This highlights the *filter* strategy’s ability to identify the most relevant features, avoiding any additional feature removal by the models embedded in the SL classifier. However, this issue should be addressed in cases where the embedded methods are implemented after a feature selection phase, such as done in Helb et al [13], as further feature removal might occur without the user’s knowledge which may affect the interpretability of the results. Hence the slight decrease in the SL performance is expected to be explained by the SL attempt to correct for a possible overfitting to the data when using RF. In this sense, these results should raise awareness concerning analysis where only RF is considered for predictive purposes, as it may lead to overfitting. Thus, the implementation of techniques such as the SL may provide more consensual results across the classifiers chosen for the predictive stage.

Comparing our results with the previous ones by Valletta and Recker [15] revealed an increase in the prediction ability of up to 14% in the best-case scenario. Not only that, but feature selection also increase the RF’s predictive ability compared to the one obtained by the same authors, an increase that ranged from 5% of in the worst-case scenario (simple antibody selection) to 12% in the best-case scenario (data dichotomization selection). These results further emphasize the impact of feature selection prior to predictive analysis. On the one hand, this step removes antibody responses with negligible effect on clinical malaria. On the other hand, this stage decreases the number of features allowing for a more thorough feature analysis increasing the chance of finding the right transformation and dichotomization for each antibody response.

Concerning the antibodies identified, we found that the antibody responses against different Merozoite Surface Proteins (MSPs) were consistently selected across the different feature selection strategies. These proteins are expressed at the parasite surface, thus, providing promising targets for malaria immunity, because they are repeatedly and directly exposed to the host humoral immune system [7, 8]. In particular, *msp2* has been associated with protection from clinical malaria in many studies and even suggested as a vaccine candidate [9–12]. For example, *msp2* has been strongly associated with protection against clinical malaria in two independent cohorts of Kenyan children [13]*. Msp4* has also been reported to have a protective effect in Kenyan children [14, 16]. High antibody levels against *msp4* constructs have been associated with reduced morbidity in a Senegalese community [17]. *Msp7* protection against malaria has also already been identified in the Kenyan population [16, 18]. Moreover, panels of antibodies comprising *msp7* have been associated with clinical protection against malaria in Kilifi, a rural district along the Kenyan coast [14]. In the same article, high antibody levels against the Erythrocyte-binding antigen-175 (*eba175*) antigen were also associated with protection from clinical malaria in children [14]. Moreover, *eba175* is associated with protection from symptomatic malaria, as demonstrated in Papua New Guinean children [15]. These findings corroborate the ability of our methodologies to identify relevant antibodies associated with protection to malaria. However, *msp10* and *h103* have not have not previously been associated with clinical malaria protection. To the best of our knowledge, this is the first study where these 2 antibodies emerge as candidates for protection against malaria. This evidence thus suggests that there are antibodies associated with protection against clinical malaria that have not yet been identified. Nevertheless, further studies are necessary to validate our findings. Finally, none of our feature selection metrics selected *msp1*, an immune response commonly associated with malaria protection and often referred to as a potential vaccine candidate. Similar findings have been reported in other studies, where *msp1* has been described to show low or no associations with exposure or protection to clinical malaria [13, 15]. These inconsistent findings further suggest the need for constructing robust feature selection strategies that could help increase reproducibility among studies. 

At this moment, the pipelines are implemented in the free R software whose scripts are publicly available for consultation and improvement. However, current implementation of the pipelines is not in the form of a stand-alone and easy-to-use package. The respective adaptation to other datasets or the deployment of the tools here developed to malaria endemic countries might require the intervention of R experts to modify the available scripts. The requirement of this specific expertise might limit the applicability of these computational tools in many malaria-endemic regions with poor human resources. Therefore, setting the computational implementation of these and other tools as a top priority is likely to help in the clinic and contribute to the development of new therapeutics and a better malaria management and control.

## Conclusions

In summary, we have implemented feature selection strategies to analyze multiple antibody data. These were developed with the idea of coupling classical, traditional statistical techniques for variable selection with popular machine learning techniques for predictive analysis. Considering the transformation of each antibody data individually these strategies represent a more flexible approach to accommodate different data patterns than those commonly described in the literature. Overall, these methodologies led to an improved classification over previous analysis based on the use of the RF alone, highlighting their potential to integrate future multi-sera pipelines.

## Data Availability

The datasets used and/or analyzed during the current study are available in this published article: Valletta, J. J. & Recker, M. Identification of immune signatures predictive of clinical protection from malaria. PLoS Comput Biol 13, e1005812 (2017). 10.1371/journal.pcbi.1005812. The R scripts generated are freely available in the following GitHub address: Immune-Stats (https://github.com/Publications/Fonseca_etal.).

## Abbreviation

AIC: Akaike’s Information Criterion
Ama: Apical membrane antigen 1
AUC: Area Under the Receiver Operating Characteristic Curve
EBA: Erythrocyte-binding antigen
ELISA: Enzyme-linked immunosorbent assay
FDR: False discovery rate
GOF: Goodness of fitness
IgG: Immunoglobulin G
LASSO: Least Absolute Shrinkage and Selection Operator
LDA: Linear discriminant analysis
Log: Logarithmic
LRM: Logistic regression model
MSP: Merozoite Surface Protein
MSRP: MSP7-related proteins
n_p_: Number of Protected individuals
n_s_: Number of Susceptible individuals
Pf: Plasmodium *falciparum*
Prt: Protected
QDA: Quadratic discriminant analysis
RF: Random Forest
ROC: Receiver Operating Characteristic
r_S_: Spearman's Correlation Coefficient
SeroTAT: Serological testing and treatment
sPLS-DA: Sparse partial least squares discriminant analysis
Sus: Susceptible
SVM: Support vector machine
SW: Shapiro-Wilk
χ^2^: Chi-square
XGB: Extreme Gradient Boosting
